# Supplementary material for: Antibiotics damage the colonic mucus barrier in a microbiota-independent manner
Source: Sci Adv. 2024 Sep 11;10(37):eadp4119. doi: 10.1126/sciadv.adp4119 (PMC11389797; doi:10.1126/sciadv.adp4119)
Supplement: Supplementary file 1 — Figs. S1 to S3 [file sciadv.adp4119_sm.pdf]

Science Advances
AAAS

Supplementary Materials for

Antibiotics damage the colonic mucus barrier in a microbiota-independent manner

Jasmin Sawaed *et al.*

Corresponding author: Shai Bel, shai.bel@biu.ac.il; Amir Erez, amir.erez1@mail.huji.ac.il

Sci. Adv. **10**, eadp4119 (2024)
DOI: 10.1126/sciadv.adp4119

This PDF file includes:

Figs. S1 to S3

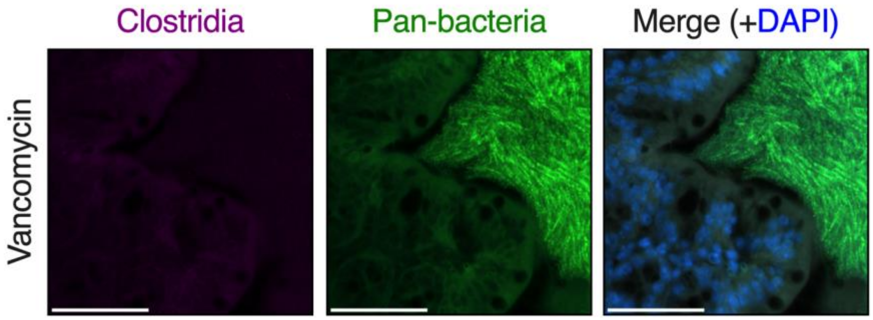

Fig S1: Vancomycin treatment depletes Clostridia from the vicinity of the host epithelium. FISH images of colonic tissues from mice treated with antibiotics as indicated and stained with the indicated probes. Scale bars, 20µm.

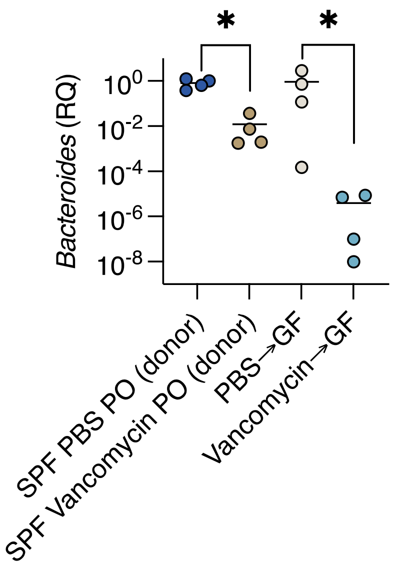

Fig S2: FMT reliably transferred the microbiota of SPF mice to GF recipients. qPCR analysis of *Bacteroides* in feces from mice treated as indicated. One-way ANOVA. *P<0.05. FMT, fecal microbiota transfer; GF, germ-free; PO, *per os*; SPF, specific-pathogen free; RQ, relative quantity.

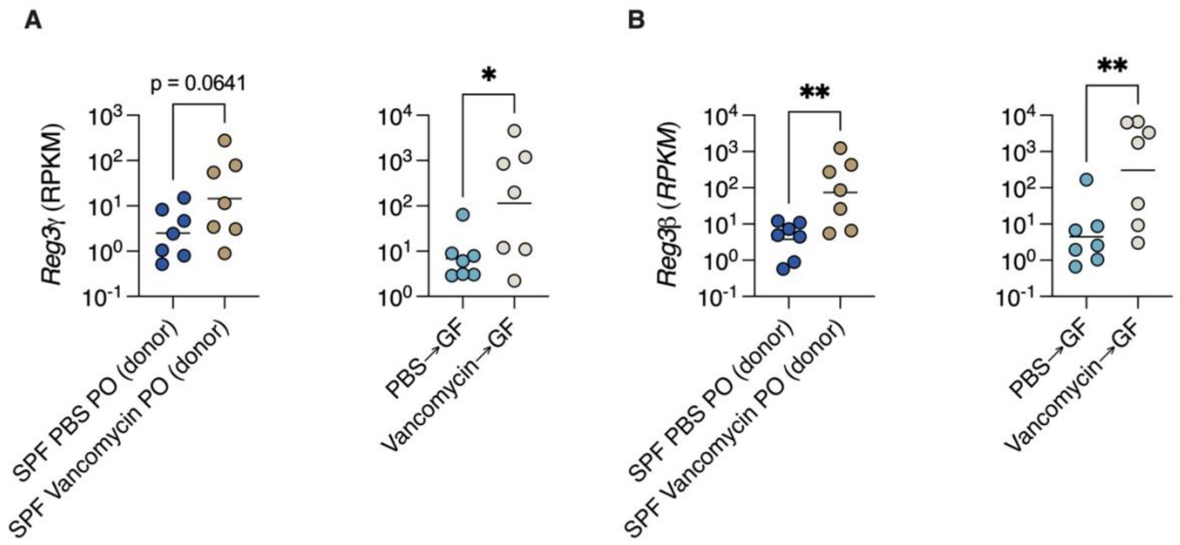

Fig S3: Antimicrobial genes are induced by vancomycin-altered microbiota. (**A-B**) Normalized reads of antimicrobial genes (**A**) *Reg3γ* and (**B**) *Reg3β* in mice treated as indicated. Each dot represents a mouse. Student's *t* test. *P<0.05, **P<0.01. RPKM, reads per kilobase per million mapped reads; GF, germ-free; SPF, specific pathogen free; PO, *per os*.
